# Factors Influencing Return to Work Among Women With Acquired Brain Injury

**DOI:** 10.1155/oti/9978566

**Published:** 2025-09-04

**Authors:** Zareena Darries, Mogammad Shaheed Soeker

**Affiliations:** Occupational Therapy Department, University of the Western Cape, Cape Town, South Africa

**Keywords:** acquired brain injury, client-centred, disability and employment, rehabilitation outcomes, vocational rehabilitation

## Abstract

**Purpose:** Research indicates that women with brain injury have a higher risk of not resuming their work roles. This study investigates the influence of sociodemographic, impairment-related and environmental factors on the return-to-work outcomes of women with acquired brain injury in Cape Metropolitan, South Africa.

**Methods:** A cross-sectional survey was conducted among 139 women aged 18–65 with acquired brain injury in Cape Metropolitan, South Africa. Participants were conveniently sampled, and the Work Rehabilitation Questionnaire was used for data collection. Data were analysed using IBM SPSS Statistics Version 26, focusing on sociodemographic, impairment-related and environmental factors influencing return to work outcomes.

**Results:** Women with acquired brain injury who participated in this study yielded a postinjury return to work rate of 61.2%. Older women were less likely to return to work (odds ratio: 0.905). Environmental support, particularly from workplace supervisors or managers, significantly enhanced RTW (odds ratio: 5.660). Marital status, impairment-related restrictions, type of vocational intervention and family support were not significant predictors of return to work.

**Conclusion:** These results highlight the necessity for multidimensional and integrative RTW programmes that address both personal and systemic barriers. Such programmes are essential to promoting sustained economic participation and improving the quality of life for women with ABI.

## 1. Introduction

Acquired brain injury (ABI) is a global public health challenge that encompasses traumatic brain injuries (TBIs) and nontraumatic injuries, such as strokes, which occur after birth and following a period of typical development [1]. TBIs affect approximately 50 million people annually, while strokes impact 12.2 million, making these conditions the primary causes of ABI worldwide [2]. ABI is a leading cause of disability, with significant implications for acute and chronic health outcomes, as well as an increased risk of neurological and neurodegenerative disorders [3]. The impact of ABI extends far beyond the immediate physical injuries, often resulting in motor impairments, chronic pain and fatigue. Cognitive challenges, such as memory loss, communication difficulties and reduced information processing capabilities, are common, as are psychosocial issues, including depression, anxiety, social isolation and reduced vocational engagement [4].

Despite advancements in medical interventions and rehabilitative technologies, managing ABI continues to remain a multifaceted challenge [2, 3]. ABI affects individuals beyond physical health, also impacting relationships, community participation and work [2, 3]. Return to work (RTW) is a key indication of a successful rehabilitation process due to its influence on economic independence, social participation and psychological well-being [5]. However, RTW is a complex process, influenced by clinical, social and gender-specific factors [6, 7]. Women with ABI frequently experience unique barriers, including fatigue, chronic pain, heightened anxiety and caregiving responsibilities, which further hinder vocational reintegration [6, 7]. Within South Africa, ABI research primarily centres around biomedical aspects, with limited literature on women's postinjury vocational outcomes [8]. While numerous existing studies have focused largely on general predictors like age and education, findings often neglect reporting on how gender, disability and socioeconomic disadvantage intersect, particularly in resource-constrained settings, such as the Western Cape in South Africa [9–11].

The limited research that does exist descriptively highlights the intersectional challenges faced by women with ABI. For example, Soeker and Darries [12] employed a qualitative methodology to explore the experiences of women with brain injuries in the Western Cape and identified barriers such as postinjury sequelae, workplace exploitation, recruitment and legislative gaps, parental responsibilities and limited access to safe transportation. However, while such qualitative studies have offered valuable insights on the lived experience of women with ABI, few studies have quantitatively examined how gender, disability and socioeconomic factors intersect collectively, influencing rehabilitation and RTW outcomes. This gap is concerning given that South Africa's sociopolitical and economic context poses significant barriers to employment for women with disabilities. For instance, women represent 8.3% of the population with disabilities compared to 6.5% for men, yet they face systemic barriers to labour market participation. In 2024, while the overall unemployment rate was 34.1%, male labour force participation stood at 75.7%, compared to 62.7% for women [13]. Additionally, people with disabilities comprise less than 1% of the workforce, far below the government's target of 2% [13]. This study is aimed at examining how sociodemographic, impairment-related and environmental factors influence the likelihood of women with ABI returning to work postinjury. The findings are expected to have significant implications for shaping vocational rehabilitation policy and practice to more effectively meet the needs of this group.

The International Classification of Functioning, Disability and Health (ICF) framework was employed to guide the researcher in defining the key variables relevant to RTW. This framework offers a holistic biopsychosocial approach to understanding health and disability, recognising that the interaction between a person's health condition, personal and environmental factors, shape and influence functioning [14]. In this study, personal factors are defined under the ICF as “the particular background of an individual's life and living,” encompassing attributes independent of health conditions [14]. These include age, marital status (classified as single, married, widowed, divorced or separated) and education level (measured as the highest level of formal education completed and categorised into primary, secondary and tertiary education). Such factors play a critical role in shaping rehabilitation and employment outcomes as they intersect with broader sociocultural dynamics and individual lived experiences. Similarly, environmental factors are defined by the ICF as “the physical, social and attitudinal environment in which people live and conduct their lives” [14]. Environmental factors included whether participants received support from family and friends, reported the presence of workplace accommodations and confirmed access to disability grants and rehabilitation programmes [14]. Additionally, it includes whether they received vocational rehabilitation interventions and their duration.

The significance of this study lies in its potential to address critical gaps in understanding the unique challenges faced by women with ABI, particularly in resource-constrained settings such as South Africa. While the global discourse on RTW outcomes has largely centred on general predictors and interventions, limited attention has been paid to the intersectionality of gender, disability and socioeconomic context. This study's focus on the Western Cape offers insights into how systemic barriers, such as legislative shortcomings, caregiving burdens and socioeconomic disparities, disproportionately affect women's vocational reintegration.

## 2. Methods

Women with ABI were conveniently sampled from the electronic databases and clinical records of the vocational rehabilitation units at two tertiary hospitals in the Western Cape, South Africa. Convenience sampling methods place primary emphasis on generalisability, thus ensuring that the knowledge gained is representative of the population of women with a clinical diagnosis of ABI [15]. A cross-sectional survey was implemented for this study by utilising the Work Rehabilitation Questionnaire (WORQ). The WORQ is a standardised instrument that assesses and records the functioning of individuals with several health conditions receiving vocational rehabilitation [16]. After ethical approval was obtained from the two hospital's ethics committees, a pilot study was conducted to evaluate the execution of the procedures and methods as well as an overall analysis and identification of weaknesses that may be addressed for use in the main study.

Only female patients who had received curative and rehabilitation services at Tygerberg Hospital (TBH) and Groote Schuur Hospital (GSH) were included in this study. Those eligible had a clinical diagnosis of ABI as ascribed under the International Classification of Diseases, Tenth Revision (ICD-10 codes) [17], including cerebral vascular accidents (CVAs), TBIs, benign brain tumours and malignant brain tumours that were not metastatic in nature. Women between 18 and 65 years were selected, as this age range aligns with the national workforce participation parameters within the South African context, ensuring that the study's findings were relevant and applicable to the working population. Furthermore, women were required to have worked in the formal labour market before their injury and to have been in a postacute brain injury stage of recovery of approximately 4–6 months. Women with acute psychiatric conditions or other medical conditions that could have influenced the sequelae of ABI were excluded.

## 3. Data Collection Instrument

The psychometric properties and evidence for the use of the WORQ as a credible instrument have been shown for its high test–retest reliability (*r* = 0.79), good internal consistency (Cronbach's alpha = 0.88), face and content validity [18]. The WORQ has been used in similar studies based in the African context and demonstrated satisfactory levels of test–retest reliability [16, 19, 20]. The questionnaire was translated into its Afrikaans version, which entailed the forward and backward translation of the questionnaire and later piloted for content and linguistic clarity [21].

## 4. Procedure

Data on demographic, health and environmental variables were collected following the standard response format of the ICF-based self-report WORQ [16]. Data collection took place over a period of 9 months. The pilot study was conducted by the principal researcher over 1 month with 13 participants who were recruited from the designated hospitals' data basis via face-to-face and telephone interviews.

Demographic variables, that is, age, marital status and education level were participant-reported and categorised by the researcher such as age years, marital status as single, married, divorced, separated or widowed. Education level was classified according to the level of schooling obtained. Likewise, health-related variables were collected and, where possible, clinical records were sourced to verify the data. These included both medical and therapeutic treatment as well as impairment-related restrictions relating to cognitive, physical, emotional or perceptual skills affecting daily functioning. Environmental variables were participant-reported and included specific support from family and friends, workplace supervisors or managers and government or private organisations. Type and duration of vocational services were collected, which included variables, that is, workplace accommodations, access to disability grants and vocational rehabilitation. The participants were able to adequately understand and complete the questionnaires in English and Afrikaans, both face-to-face and telephonically. It was found that the estimated time frame required for the administration of the questionnaire was a maximum of 10 min per survey during a face-to-face interview and 15 min via a telephonic interview.

The main study commenced after the successful completion of the pilot study. Interviews were conducted by the principal researcher. Additionally, an independent occupational therapist was later recruited and trained in administering the instrument to provide assistance when needed. Information sheets outlining the research and consent forms were provided to 190 participants who met the study's inclusion criteria. Of these, 177 participants provided consent to take part. To ensure continuity and data consistency, the researcher employed the same administration methods used during the pilot study. A total of 156 completed questionnaires were returned, yielding a response rate of 89.2% (*n* = 156/177). Then, 17 questionnaires were incomplete, resulting in a final response rate of 80% (*n* = 139/177). In this study, listwise deletion was employed to address missing data, which ensured that all included cases have complete data for all variables analysed.

## 5. Analysis

Data were entered and analysed using IBM SPSS Statistics Version 26 [22]. The demographic, health and environmental-related characteristics as well as the RTW of the study participants were summarised using descriptive statistics of measures of central tendency, frequency and percentages [23]. Inferential statistics were used to determine whether sociodemographic, impairment-related restrictions and environmental factors predicted return to work among women with ABI.

### 5.1. Description and Operationalisation of Variables

#### 5.1.1. RTW Status

RTW in this study refers to the resumption of work within the competitive labour market, encompassing formal employment (e.g., salaried positions), informal work (e.g., freelance or casual labour) and self-employment. To contextualise this definition, it aligns with prior research, which describes RTW as not only returning to preinjury employment but also adapting to new roles that align with postinjury capacities [24]. This operationalisation captures the diversity of employment outcomes post-ABI, including modified work tasks or hours. Impairment-related restrictions were recorded as “Yes”; the presence of cognitive, perceptual, physical or emotional difficulties was collapsed into a single category, or “No”, for the absence of impairments. The sociodemographic and environmental variables were also operationalised for analysis. The participants' ages were self-reported in years, while marital statuses were categorised as single, married, separated, divorced or widowed. Level of education was categorised as primary, secondary and tertiary education. Treatment received was recorded as a binary, “Yes” or “No” in response to the participants' reported medical or therapeutic intervention postinjury. Vocational rehabilitation was categorised into three groups, that is, vocational intervention (combining cognitive, physical, case management, vocational training and work adaptation services), work evaluation and no intervention.

### 5.2. Inferential Statistical Analysis

The variables that predicted RTW among women with ABI were determined using a binary logistic regression analysis [24].

Then, 10 variables were tested in the equation. The key dependent variable, that is, RTW status, was categorised as have not returned to work (No) and returned to work (Yes). Nine key independent variables were set in the model, that is, age, marital status, educational level, treatment received, impairment-related restrictions, type of vocational intervention received, family and friends' support, workplace supportand support from government or private organisations. The logistic regression analysis was executed on the variables and the level of statistical significance was set at the 95% confidence level, with a confidence interval of 0.05.

## 6. Results

The results are presented according to this study's objective. Then, 52% of the participants were aged between 20 and 40 years, with a mean age of 40.17 (SD = 10.56, range = 43). Over 62% (62.6%) of participants were married. The majority had reached and not necessarily completed secondary education (70.5%), while 18.7% had only completed primary education, and 10.8% obtained a tertiary education. Then, 75% of participants reported receiving medical or therapeutic intervention, and 86.4% experienced impairment-related restrictions that affected their ability to perform certain daily tasks and activities. More than half of the participants (57.1%) had not received vocational rehabilitative intervention, while 20.3% had undergone work evaluation and 22.6% had received cognitive, physical or work adaptation interventions.

### 6.1. Supports Towards Work Resumption

**Figure 1:**
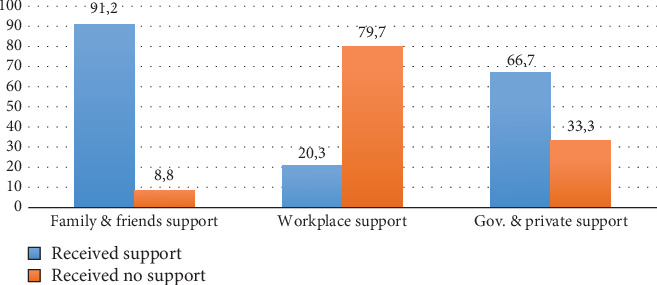
Environmental supports to RTW.

Most of the participants (91.2%) received support from their family members and friends in the form of emotional, physical and financial aid post-ABI. More than half of the participants (66.7%) indicated having received support from government and private organisations, which included government financial aid, while the private organisations offered paid learnership opportunities that provided training, work practice opportunities and job placement (see Table 1). However, most (79.7%) of the study participants indicated having received no support from supervisors or managers in the work environment post-ABI (Figure 1).

### 6.2. Variables Predicting RTW (Binary Regression Analysed)

Binary regression is a statistical method used to model binary outcome variables—in this case, whether participants returned to work (RTW; coded as 1) or did not (coded as 0). It estimates the likelihood (or odds) of a specific outcome occurring based on predictor variables, such as sociodemographic, health status and environmental support factors. This method is particularly appropriate for the research question, as it allows for examining the relative influence of multiple predictors on RTW while controlling for potential confounders. The model is significant, *χ*^2^(11, 139) = 38.97, *p* < 0.001 (Table 2). Between 24.5% (Cox and Snell *R*^2^) and 33.2% (Nagelkerke *R*^2^) of the variance in whether sociodemographic, health status and environmental support factors of women with ABI are explained by the model. The Hosmer and Lemeshow test was significant, *χ*^2^(11, 139) = 15.73, *p* = 0.046, with a PAC of 77.7%.

Age predicted RTW, Wald *χ*^2^ = 16.822, *p* < 0.05. Older women were less likely to RTW compared with younger women (OR = 0.905, 95%CI = 0.863, 949). The OR of 0.905 for age suggests that with each additional year of age, the odds of RTW decrease by 9.5%. Level of education marginally predicted RTW, Wald *χ*^2^ = 3.558, *p* = 0.059. The OR of 7.617 suggests that women with tertiary education are approximately 7.6 times more likely to RTW compared to those without tertiary education. However, due to the *p* value being slightly above the significance level, the result is not statistically significant at the 5% level, and there is a degree of uncertainty in the estimate. Environmental support predicted RTW, Wald *χ*^2^ = 4.385, *p* = 0.036. Those who received support from their managers or supervisors in the workplace were about five times more likely to RTW compared with those who received support from family and friends (OR = 5.660, 95%CI = 1.117, 28.666). The OR of 5.660 for workplace support underscores the critical role of employer-driven interventions in facilitating RTW.

Marital status failed to predict RTW, Wald *χ*^2^ = 0.138, *p* > 0.05. The probability for those who were married and those who were single had similarly low likelihoods of RTW (OR = 1.186, 95%CI = 0.482, 2.198). Likewise, treatment failed to predict RTW, Wald *χ*^2^ = 0.033, *p* = 0.856 (OR = 1.110, 95%CI = 0.359, 3.429). Impairment-related restrictions failed to predict RTW, Wald *χ*^2^ = 1.386, *p* = 0.239 (OR = 2.484, 95%CI = 0.546, 11.294) and type of vocational intervention failed to predict RTW, Wald *χ*^2^ = 0.274, *p* = 0.610 (OR = 0.754, 95%CI = 0.262, 2.169). Furthermore, support from family failed to predict RTW, Wald *χ*^2^ = 0.054, *p* = 0.816, indicating those who had received family support and those who had not received support have a similarly low likelihood of RTW (OR = 0.822, 95%CI = 0.158, 4.267).

## 7. Discussion

The results of the current study showed a RTW rate of 61.2% for women with ABI. The binary approach allowed for a clear and logical outcome; however, it may have overlooked specific differences in the type and sustainability of the work. The inclusion of formal, informal and self-employment used as the operational definition of RTW status is aimed at reflecting the varied work experience of women postinjury; however, it may also have influenced the RTW rate observed in this study [25]. The results remain noteworthy when compared to the general labour force participation of women (62.7%) as reported in the City of Cape Town's Socio-Economic Profile of 2017 [26]. The results showcase the determination and resilience of women with ABI to return to the workforce despite the challenges posed by their injuries. However, the high rate amongst participants may also be influenced by the need for employment, especially in resource-constrained settings such as South Africa. The City of Cape Town's labour force participation of women shows their vital role in contributing to household income [26]. Furthermore, limited financial aid or sustainable state disability grants often compels women with disabilities to work while not necessarily being ready to RTW. The ICF framework emphasises that contextual factors interact with individual health conditions and may also influence vocational outcomes [14]. Therefore, the RTW rate of the current study's findings could reflect that women with ABI may RTW while not necessarily being fully recovered from their injury or working in the best conditions, but rather for livelihood and survival needs.

### 7.1. Sociodemographic Factors Associated With RTW

#### 7.1.1. Age

The results revealed a significant association between age and RTW, demonstrating that women (40–50 years) with ABI were less likely to return to competitive employment post-ABI. These findings coincide with several studies where age was identified as a major predictor of RTW success, with individuals under the age of 40 years generally faring better than those over the age of 40 [27]. Corrigan et al. [28] found that in comparison with men, women with ABI were more likely to decrease hours or be unemployed, whereas the findings in other studies run counter to existing literature where the age of women with ABI was not found to be an RTW risk marker [11, 29, 30]. However, divergences in research findings about person-related factors affecting RTW for individuals with ABI could likely be attributed to the different studies' methodological designs, generalisability or recruitment bias linked to demographics and the legal and social framework of the research setting or country [31, 32]. Given the inherently lower employment rate for women in general in the Cape Metropolitan, Western Cape, the person-related factors and the socioeconomic context of the study setting could be an indicator for poorer RTW rates for older women with ABI. The likelihood of increased dependence on state disability/social security grants could further contribute to disabling circumstances for older women with ABI in the current study context. This coincides with Etuknwa et al. [33], who found that women with disabilities from low- and middle-income countries (LMICs) frequently experience pressure to return to informal or unsecure work due to poverty and social insecurity [33]. Therefore, it would be important to adopt RTW interventions that recognise the needs and factors related to age and stage, as well as patterns across activity and occupational participation of women with ABI [34]. However, it is critical to delve deeper into the interaction between age and other sociodemographic variables, such as educational background and socioeconomic status, and to explore the potential mechanisms underlying these trends. This finding also underscores the need for age-sensitive vocational rehabilitation programmes, such as offering flexible work arrangements or targeted retraining opportunities for older women.

#### 7.1.2. Marital Status

While being married has shown to be a positive predictor of RTW for women with ABI [28], in this study, being married was not associated with RTW. Gendered expectations in many societies position men as primary wage earners, and this norm may create flexibility for married women with ABI to withdraw from the labour force or reduce working hours, particularly if household income is not significantly impacted [33]. On the other hand, unmarried women, who may bear sole financial responsibility for their households, might face greater pressure to re-enter the workforce, albeit with challenges stemming from ABI-related impairments [34]. In lower income contexts or where economic security is uncertain, married women may still need to contribute to household income, reducing the likelihood of reduced employment. In contrast, in high-income settings, a supportive spouse's income may enable greater flexibility for recovery or alternate roles within the household. Unmarried women, particularly those with children, may encounter additional burdens as both caregivers and breadwinners, complicating their RTW trajectory. Such dynamics suggest that the effect of marital status on RTW may be context-dependent, influenced by factors such as spousal employment, income and cultural norms.

In Corrigan et al. [28], it was found that decreased employment was most evident for married women with ABI and they were more likely to reduce hours or stop working compared to single women. Whereas in Donker-Cools et al. [30], it was found that not being married appeared to be negatively associated with RTW after ABI, specifically the odds of being unemployed versus being employed were 1.57 times greater for unmarried versus married women. According to Matérne et al. [7], the interface between gender and marital status may result from men being more likely to be the primary wage earner in the family, which could likely provide a better opportunity for women with ABI to either stop working or work fewer hours. However, for those women who are not married, it is important to consider the occupational and breadwinner roles in the family unit that could reflect different dynamics based on their social and economic situations, particularly in resource-constrained settings, such as the Western Cape, South Africa [7].

#### 7.1.3. Education

Although the relationship between tertiary education and RTW is not statistically significant at the 5% level, it indicates that there may be a potential connection worth further inquiry. The transformative impact of higher education on employment outcomes is well documented and serves a key role in determining RTW success, potentially mitigating some age-related disadvantages [28]. Some studies revealed that higher education levels are associated with improved access to white collar jobs that may offer greater flexibility and accommodations for individuals with ABI. On the contrary, older women with lower educational qualifications are more likely to be employed in physically demanding roles, where cognitive or physical impairments are less easily accommodated [28]. The current study did not collect comprehensive data on the specific job types or demands that participants returned to postinjury, nor did it analyse whether cognitive impairments were easier or difficult to accommodate in certain work roles. However, this dynamic could create a compounded disadvantage for older women in blue-collar sectors, as these roles often lack robust RTW policies or the capacity for task modification, especially within the current study setting.

Several studies have supported the notion that a higher educational level is more positively associated with RTW [32, 35]. In Matérne et al. [7], findings revealed that 45% of the individuals with a university degree returned to work while only 32% of those with compulsory school education returned to work. The educational levels for the population of the Cape Metropolitan in 2016 established that only 34% of the population obtained a secondary school certification and 14.4% obtained a higher education certification [26]. Indicative of the abovementioned educational statistics, the majority (70.5%) of this study's participants only reached (and not necessarily completed) a secondary level education. It should be noted that the definition used to operationalise RTW status in this study likely captured participants who returned to their preinjury work roles as well as those who returned to new or more conducive work contexts that aligned more with their postinjury abilities; additional data might help clarify this relationship. Furthermore, policy could focus on improving access to education and vocational training for women with ABI while RTW interventions strengthen inclusiveness of those with different levels of education. Lastly, to enhance effectiveness, workplace education and skills transfer should be individually tailored during the rehabilitation process [32].

### 7.2. Impairment-Related Factors Associated With RTW

Although the majority of participants (86.4%) reported experiencing impairment-related restrictions that affected their ability to perform daily tasks and activities, the current study did not find a significant association between RTW and the presence of cognitive, perceptual, physical or emotional restrictions post-ABI. The qualitative study findings by Soeker and Darries [12] revealed that RTW challenges for women with brain injury included postinjury sequelae that impacted on their ability to adapt to their premorbid worker roles. Research findings that qualitatively report sex and gender differences after brain injury could demonstrate important first-hand differences that are shaped by gender roles, identities and norms [33]. In view of the context of the current study, the possible reason for the insignificance could be that women are often obliged to work in order to contribute or support the household irrespective of possible health-related factors. Furthermore, the broad categorisation of impairments (e.g., cognitive, physical and emotional) may obscure nuanced differences in how specific impairments impact RTW. For instance, cognitive impairments such as memory deficits or executive dysfunction may have a more profound effect on job performance in high-skill occupations, whereas physical impairments might be more relevant in physically demanding jobs. Future research could explore the differential impact of impairment types on RTW across occupational contexts.

### 7.3. Environmental Factors Associated With RTW

#### 7.3.1. Family and Friends' Support

With supportive environments, some studies have shown that support from family is important for patients with a brain injury during RTW [34]. The results of the current study revealed that support from family and friends did not predict RTW for women with ABI. A possible explanation could have been that such support may have assisted with emotional adjustment and coming to terms with the injury; however, it might not have been sufficient to address practical RTW assistance. Several literatures support the notion that social support, including family members, friends and community members, were viewed as necessary for successful RTW [34]. In Ownsworth [34] as part of a client-centred RTW approach, the family support system and their involvement in work-related decisions were listed as important components in reorganising the home and family routine whilst strengthening their work readiness and commitment to work. However, in some contexts, cultural factors and practices could influence RTW, where family prioritise caring for and encourage recovery, while motivation and support for RTW might be less focused on vocational goal attainment [29].

#### 7.3.2. Workplace and Governmental/Private Organisations' Support

The results of this study further showed that participants who received support from managers or supervisors in their workplaces were about five times more likely to RTW compared with those who received support from family and friends. Although age was found to be a significant predictor of RTW in this study, the stronger RTW association of workplace support highlights the essential role of environmental enablers. This is not surprising considering the vast empirical evidence on the success rate of RTW of individuals with ABI when there is a supportive workplace, inclusive organisational culture and positive social relations with employers and co-workers. Workplace accommodations and support mechanisms may mitigate the impact of impairment-related restrictions on RTW [36]. Flexible working conditions, job modifications, assistive technologies and supportive employers can enable individuals with cognitive, physical or emotional impairments to reintegrate into the workforce despite their limitations [37]. Therefore, women with ABI would benefit from a RTW programme that applies approaches that equally address metacognitive and contextual factors, including creating supportive and enabling environments both within the home and work context [34].

#### 7.3.3. Rehabilitative Support

The current study did not find a significant association between those participants who received medical or therapeutic treatment and RTW. Similarly, the type of vocational rehabilitation received by these study participants was not associated with RTW. Differences in findings could be attributed to the South African context, where vocational rehabilitation services are often constrained by limited resources and a focus on meeting disability grant eligibility criteria. Unlike the comprehensive systems observed in European settings, South African vocational rehabilitation primarily operates within the healthcare system with restricted inter-sectoral integration [38]. This narrow scope undermines the quality of rehabilitation and hinders the development of comprehensive RTW programmes [38, 39]. Similarly, Forslund et al. [37] found that institutional support such as the number of rehabilitation services was not a significant predictor in their study but the need for well-coordinated healthcare and rehabilitation services was deemed essential.

Furthermore, findings of Cancelliere et al. [27] highlight that successful RTW interventions are often characterised by work-specific training, structured RTW planning, workplace interventions, stakeholder engagement and the involvement of a dedicated case coordinator. These elements are essential when developing RTW programmes in order to enhance effective and economic sustainability for women with ABI in South Africa.

## 8. Study Limitations

A limitation of this study is noted in the sampling setting, where participants were recruited from the vocational rehabilitation units of two tertiary hospitals, where individuals are typically admitted based on an expressed vocational goal or the potential to RTW. This may have introduced selection bias, as women could have likely been more motivated to RTW, potentially contributing to the study's high RTW rate. Therefore, this study might not reflect the broader population of women with ABI.

Additionally, due to a low attendance rate of female patients who met the inclusion criteria on the specified clinic dates at the study sites, fewer face-to-face interviews took place. Therefore, telephonic interviews were conducted with the majority of the study participants. Telephonic interviews were costly and limited the response time, which could have affected the qualitative response options from the survey respondents [40].

## 9. Conclusion and Recommendations

The study highlights that successful RTW outcomes for women with ABI are significantly influenced by sociodemographic factors such as age and education level, as well as environmental support systems. These findings contribute to the broader literature by emphasising the intersection of personal and systemic factors in shaping RTW trajectories, particularly in resource-constrained settings. The results also underline the need for vocational rehabilitation programmes to be multidimensional, addressing these critical variables through tailored interventions that reflect the unique challenges faced by women with ABI.

The practical implications of this study suggest the importance of integrating age-appropriate training, education-focused rehabilitation and environmental accommodations into RTW programmes. Policymakers, rehabilitation specialists and employers must collaborate to develop inclusive strategies that support women with ABI in reintegrating into the workforce. This includes funding rehabilitation plans, workplace adaptations and fostering inter-sectoral partnerships to address systemic barriers.

## Figures and Tables

**Table 1:** Demographics, health and rehabilitative characteristics of respondents (*n* = 139).

**Variable**	**N**	**%**
Age (years)		
≤ 40	73	52.5
41–50	40	28.9
51–65	26	18.8
Marital status		
Single	52	37.4
Married	87	62.6
Education level		
Primary school	26	18.7
Secondary school	98	70.5
Tertiary	15	10.8
Received medical/therapeutic treatment		
Yes	113	81.2
No	26	18.7
Impairment-related restrictions		
Yes	108	77.7
No	31	22.3

*Note:* Single includes individuals who were never married, divorced/separated or widowed. Tertiary education includes college, university and postgraduate qualifications. Impairment-related restrictions include cognitive, perceptual, physical and emotional.

**Table 2:** Summary of logistic regression analysis for variables predicting RTW (*n* = 139).

**Predictors**	**B**	**SE**	**Wald ** **χ** ^2^	**p**	**e** ^**B**^	**95% CI**
Age	−0.099	0.024	16.822	0.000	0.905	[0.863, 0.949]
Marital status	0.171	0.459	0.138	0.710	1.186	[0.482, 2.198]
Education			4.122	0.127		
Secondary	0.027	0.574	0.002	0.963	1.027	[0.333, 3.165]
Tertiary	2.030	1.076	3.558	0.059	7.617	[0.924, 62.803]
Treatment received	0.104	0.576	0.033	0.856	1.110	[0.359, 3.429]
Impairment-related restrictions	0.910	0.773	1.386	0.239	2.484	[0.546, 11.294]
Type of vocational rehabilitation			0.819	0.664		
Work evaluation	−0.612	0.676	0.819	0.365	0.542	[0.144, 2.039]
No intervention	−0.282	0.539	0.274	0.601	0.754	[0.262, 2.169]
Family support	−0.196	0.840	0.054	0.816	0.822	[0.158, 4.267]
Workplace support	1.733	0.828	4.385	0.036	5.660	[1.117, 8.666]
Government and private support	0.165	0.496	0.111	0.739	1.180	[0.446, 2.3121]
*χ* ^2^		38.97				
Df		11				
%		77.7				

## Data Availability

The data that support the findings of this study are available on request from the corresponding author. The data are not publicly available due to privacy or ethical restrictions.
